# GoldenFish: a rapid and efficient system to customize constructs for zebrafish transgenesis

**DOI:** 10.1093/jmcb/mjac075

**Published:** 2022-12-24

**Authors:** Zhanmei Jiang, Lirong Huang, Jieqiong Zhao, Yanfeng Li, Jianlong Ma, Rui Ni, Qifen Yang, Lingfei Luo, Yun Yang, Jingying Chen

**Affiliations:** Institute of Developmental Biology and Regenerative Medicine, Southwest University, Chongqing 400715, China; Institute of Developmental Biology and Regenerative Medicine, Southwest University, Chongqing 400715, China; Institute of Developmental Biology and Regenerative Medicine, Southwest University, Chongqing 400715, China; Institute of Developmental Biology and Regenerative Medicine, Southwest University, Chongqing 400715, China; Institute of Developmental Biology and Regenerative Medicine, Southwest University, Chongqing 400715, China; Institute of Developmental Biology and Regenerative Medicine, Southwest University, Chongqing 400715, China; Institute of Developmental Biology and Regenerative Medicine, Southwest University, Chongqing 400715, China; Institute of Developmental Biology and Regenerative Medicine, Southwest University, Chongqing 400715, China; Institute of Developmental Biology and Regenerative Medicine, Southwest University, Chongqing 400715, China; Institute of Developmental Biology and Regenerative Medicine, Southwest University, Chongqing 400715, China; University of Chinese Academy of Sciences (Chongqing), Chongqing Institute of Green and Intelligent Technology, Chinese Academy of Sciences, Chongqing 400714, China

Transgenesis, which inserts exogenous DNA into animal genomes, is a widely used technique. Traditionally, the constructs for transgenesis are generated by step-by-step subcloning of DNA fragments, which requires multiple steps depending on the construct complexity. To overcome the limitation, advanced tools such as Gateway cloning (Hartley et al., 2000; Kwan et al., 2007), In-Fusion cloning (Sleight et al., 2010), and Gibson assembly (Gibson et al., 2009) have been developed. However, due to their ligation characteristics, no systematic method for transgenesis has been developed. ‘Golden Gate’ cloning first appeared in 2008, which is a widely used DNA assembly method (Engler et al., 2008, 2009). Here, we take zebrafish transgenesis as an example and develop a standardized system called GoldenFish, which is based on Golden Gate cloning. It can customize transgenic constructs in one step and can be applied to multiple types of transgenesis such as one gene driven by one promoter, multiple genes driven by one promoter, and multiple genes respectively driven by multiple promoters, significantly reducing working time.

## Generation of P–G–T construct: one gene driven by one promoter

**Figure 1:**
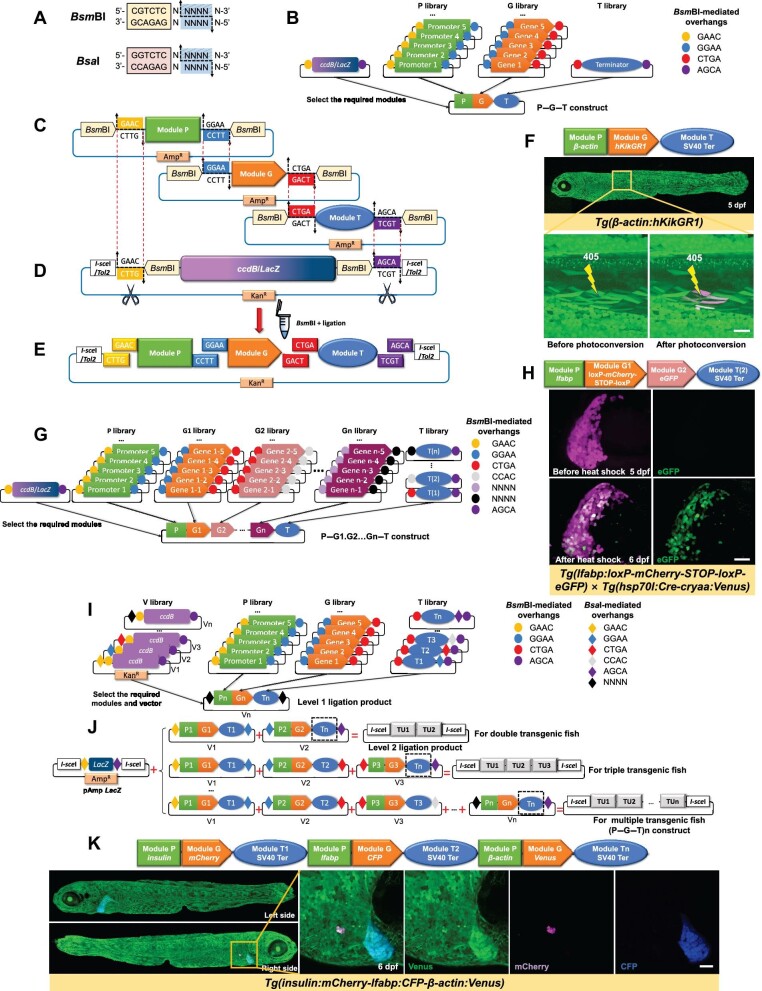
Overview of GoldenFish for generating different types of transgenic constructs. (**A**) The *Bsm*BI/*Bsa*I restriction site contains a 6-bp recognition site (yellow/pink) and a cleavage site outside the recognition site (blue). (**B**) Generation of P–G–T construct. The small circles represent *Bsm*BI-mediated overhangs. (**C**) Three modules are cloned into T-vectors conferring ampicillin resistance (Amp^R^) and flanked by inward-facing *Bsm*BI sites, which expose the designated 4-nt overhangs (colored boxes) after *Bsm*BI digestion. The 3′ overhangs of the upstream module must be compatible with the 5′ overhangs of the subsequent module for correct assembly. (**D**) GoldenFish recipient vector containing *ccdB* or *LacZ* cassette exposes overhangs matching with the 5′ overhang of Module P and the 3′ overhang of Module T. The *ccdB* or *LacZ* cassette is cut off from the vector, enabling negative or blue–white screening. (**E**) The P–G–T construct is assembled in a user-specified order. Only a predetermined construct eliminating all *Bsm*BI sites can stably exist. (**F**) *Tg(β-actin:hKikGR1)* confocal image shows the whole body expressing light conversion fluorescent protein hKikGR1. Under the irradiation of a 405-nm laser, the focused region was photo-converted from green to red. Scale bar, 50 μm. (**G**) Generation of P–G1.G2…Gn–T construct. The final construct has one gene selection T(1), two gene selection T(2), etc. (**H**) Confocal images of *Tg(lfabp:loxP-mCherry-STOP-loxP-eGFP)* identified with the background of *Tg(hsp70l:Cre-cryaa:Venus)*. After heat-shock, Cre-mediated recombination resulted in most of the mCherry^+^ hepatocytes beginning to express eGFP. Scale bar, 50 μm. (**I**) Step 1: modules generate complete TUs flanked by newly generated *Bsa*I sites (NNNN) and get kanamycin resistance (Kan^R^) via a *Bsm*BI Golden Gate reaction. Diamonds represent *Bsa*I-mediated overhangs. (**J**) Step 2: TUs are further assembled into (P–G–T)n construct via a *Bsa*I Golden Gate reaction. TUs with corresponding numbers select vectors and terminators with corresponding numbers. For example, TU1 selects Vector 1 and T1. But the last TU always selects Tn (black dotted line box). (**K**) A triple-transgenic line was generated by GoldenFish, which expresses different fluorescent proteins in the pancreatic β cells, liver, and whole body simultaneously. Scale bar, 50 μm.

Type IIS restriction enzymes are the core element of Golden Gate cloning. *Bsm*BI and *Bsa*I are chosen in our system. Generating one promoter-driven transgenic fish only needs *Bsm*BI, which recognizes asymmetric DNA sequences 5′-CGTCTC-3′ and cleaves outside of this site, leaving a 4-nt single-stranded overhang that can be designed artificially (Figure 1A).

The GoldenFish system modularizes all components of the construct and then composes different libraries according to their functions, including promoter library (P library), target gene library (G library), and terminator library (T library). Each module resembles a ‘Lego block’ with two inward-facing *Bsm*BI sites flanked. The overhangs mediated by *Bsm*BI are unique four bases that are designed to provide compatibility from one module to the next to ensure the correct orientation. In the P and G libraries, modules from the same library can produce the same overhangs, which allow them to be interchanged at will (Figure 1B and C).

Adding new modules is easy. Desired sequences can be amplified by primers added with a 5′ extension containing a *Bsm*BI restriction site. For example, to amplify Module P, we add ‘CGTCTCNGAAC’ to the forward primer and ‘CGTCTCNTTCC’ to the reverse primer. For Modules G and T, we just change the overhangs to GGAA and CTGA. Then, the purified polymerase chain reaction (PCR) products are cloned into pGEM-T vectors for long save and applied to the following Golden gate reaction. After a one-pot restriction–ligation procedure, modules can be inserted into the recipient vector in a determined order with the GAAC, GGAA, CTGA, and AGCA overhangs, called P–G–T construct (Figure 1C–E). Primers are provided in Supplementary Table S1.

To screen the correct assembly, three recipient vectors, pKan-*ccdB*, pKan-I-*LacZ*, and pKan-T-*LacZ*, were modified from commercial plasmid pDsRed1-N1 (Figure 1D; Supplementary Figure S1A–C). In addition, to improve connection efficiency, the terminator can be inserted into pKan-I-*LacZ* as a special vector for P–G–T construct only, named pKan-Ter-*LacZ* (Supplementary Figure S2A). These vectors contain a convenient negative selectable marker *ccdB* (Bernard and Couturier, 1992) or a blue–white screening marker *LacZ* flanked by two outward-facing *Bsm*BI sites. Under *Bsm*BI digestion, the linearized vectors expose overhangs compatible with the 5′ overhang of Module P and the 3′ overhang of Module T. The recipient vectors confer kanamycin resistance (Kan^R^), while the modules confer ampicillin resistance (Amp^R^). After resistance and negative/blue–white screening, a correct assembly that replaces the *ccdB* or *LacZ* cassette can be screened out. *I-Sce*I or mini*Tol2* is inserted upstream and downstream of the *ccdB* or *LacZ* cassette, which allows freely choosing meganuclease or transposase system.

Using this method, three stably inherited transgenic fish were generated, by P(ubiquitously expressed promoter *β-actin*)–G(a humanized photoconvertible fluorescent protein hKikGR1)–T(SV40 terminator) construct for *Tg(β-actin:hKikGR1)* (Figure 1F), or by P(heat-shocked promoter *hsp70l*)–G(fluorescent protein mCherry and Venus)–T(SV40 terminator) construct for *Tg(hsp70l:mCherry)* and *Tg(hsp70l:Venus)* (Supplementary Figure S2C and D). Their expression efficiencies are listed in Supplementary Table S2.

## Generation of P–G1.G2…Gn–T construct: multiple genes or sequences driven by one promoter

In this case, G2–Gn libraries are added after the G library to provide other genes or sequences (Figure 1G; Supplementary Figure S3A). Meanwhile, library P, library G (here G1), and library T can still be used. T library plays two roles, providing a terminating function and serving as a linker offering different 5′ overhangs to connect the last Gn and recipient vector. If other sequences, such as tags or linkers, are needed between two genes, they can be generated in front of Module Gn by PCR or generated as an independent module.

Here, we illustrate the situation with one promoter driving two genes or sequences as an example to generate two stably inherited transgenic lines, *Tg(lfabp:loxP-mCherry-STOP-loxP-eGFP)* and *Tg(lfabp:loxP-STOP-loxP-H2B-eGFP)*, driven by *lfabp* (liver fatty acid binding protein) promoter. They were identified by out-cross with *Tg(hsp70l:Cre-cryaa:Venus)*. After heat-shock, Cre-mediated recombination resulted in the excision of the floxed cassette (Figure 1H; Supplementary Figure S3C). This *lfabp* promoter has a positive rate of 70%–75% in founders and ∼40% of them can inherit the traits to the F1 generation.

## Generation of (P–G–T)n construct: multiple genes respectively driven by multiple promoters

In addition to *Bsm*BI, *Bsa*I is used simultaneously (Figure 1A). Assembly of (P–G–T)n constructs consists of two steps. First, modules from each library are ligated to generate complete transcription units (TUs) (Figure 1I). And these TUs can be further assembled into multi-TUs (Figure 1J). In the first step, we design a set of recipient vectors with different *Bsa*I cutting sites, which provide different 5′ *Bsa*I-mediated overhangs in the front of the *ccdB* cassette, and a set of corresponding Module T, which provides different 3′ *Bsa*I-mediated overhangs in the back. By *Bsm*BI cutting and T4 ligation, all TUs are flanked by two *Bsa*I sites, and each 4-nt 3′ *Bsa*I-mediated overhang of the first TU can be complementary to the 5′ overhang of the next TU (Figure 1I). In the second step, we generate a new recipient vector called pAmp *LacZ* with Amp^R^ (Supplementary Figure S1D). It has *I-Sce*I and *Bsa*I sites flanking *lacZ*, which can expose overhangs compatible with the 5′ overhang of the first TU and the 3′ overhang of the last TU. Through resistance and blue–white selection, TUs can be further assembled into multi-TUs (Figure 1J).

As a proof of principle, we used this method to generate some multi-transgenic fish. Tissue-specific single TUs were first generated through *Bsm*BI-mediated reaction. Then, these multi-promoters-driven TUs were further assembled through *Bsa*I-mediated reaction leading to simultaneously expressing different fluorescent proteins in different organs (Figure 1K; Supplementary Figure S4). Their expression efficiencies are listed in Supplementary Table S2.

## Generation of fusion module

It is necessary to generate a fusion module when encountering the following situations: (i) a module is very complex and needs to connect multiple fragments that are often used as a whole; (ii) several fragments need to form a seamless open reading frame; and (iii) some bases in the module require mutation.

Since the fusion module should be general-purpose with basic modules, they must have two *Bsm*BI recognition sites that generate rule-compliant overhangs and confer ampicillin resistance. Therefore, pAmp *LacZ* is chosen as the recipient vector. All fragments adding *Bsa*I sites are amplified by PCR. In some cases, overhangs should be selected in the reading frame to form a seamless connection. Meanwhile, *Bsm*BI sites are added by primers upstream of the first fragment and downstream of the last one, with overhangs (marked with 1234/5678) selected according to the library where the modules are located (Supplementary Figure S5A). As an example, we generated a fusion module of *mCherry-P2A-H2B-eGFP* and placed it downstream of liver-specific promoter *lfabp* (Supplementary Table S1 and Figure S5B).

One disadvantage of our system is that the internal type IIS restriction enzymes (*Bsa*I and *Bsm*BI) must be eliminated in all modules. This problem can be solved by the fusion module way. Mutation of *Bsa*I or *Bsm*BI sites can be extended to any base that needs modification. Primers carry overhangs that overlap mutation sites and introduce a synonymous mutation in the protein-coding sequence (Supplementary Figure S5C). But in noncoding regions, it is necessary to consider whether the mutation will change the original function.

We made some supplements as the initially created libraries. Because it is a start-up, the number of components is limited, but it can be gradually enriched during subsequent use (Supplementary Table S1). The verifications of these supplements are shown in Supplementary Figure S6.

This is a systematic method covering all types of transgenic fish achieving 95%–100% connection efficiency. The ‘Lego block’ modules bring the advantages that the construct can be customized and easily fulfilled in one-step. In particular, it can easily generate the construct including multiple transgenes at one time. Our system, including all the modules, is applicable to mammals and other organisms.

*[We thank Wensheng Wei (Peking University) for providing plasmid and Qingliang Zou for technical assistance. This work was supported by the National Natural Science Foundation of China (32270859, 32000576, and 32192400), the National Key R&D Program of China (2021YFA0805000), and the Natural Science Foundation of Chongqing (cstc2020jcyj-msxmX0882). Z.J., J.C., Y.Y., and L.L. designed the experimental strategy and wrote the manuscript. Z.J., L.H., J.Z., and Y.L. generated the modules and recipient vectors. Z.J., L.H., R.N., and Q.Y. generated and screened out transgenic fish. J.M. generated the Tg(hsp70l:Cre-cryaa:Venus) line. Z.J. performed all the other experiments in the study.]*

## Supplementary Material

mjac075_Supplemental_File
