# Supplementary material for: GoldenFish: a rapid and efficient system to customize constructs for zebrafish transgenesis
Source: J Mol Cell Biol. 2022 Dec 24;14(12):mjac075. doi: 10.1093/jmcb/mjac075 (PMC10120350; doi:10.1093/jmcb/mjac075)
Supplement: mjac075_Supplemental_File [file mjac075_supplemental_file.pdf]

Supplementary information

GoldenFish: a rapid and efficient system to customize constructs for zebrafish transgenesis

Zhanmei Jiang,[1] Lirong Huang,[1] Jieqiong Zhao,[1] Yanfeng Li,[1] JianLong Ma,[1] Rui Ni,[1] Qifen Yang,[1] Lingfei Luo,[1] Yun Yang,[1,*] and Jingying Chen[1,2,*]

List of supplementary information

Figure S1

Figure S2

Figure S3

Figure S4

Figure S5

Figure S6

Table S1

Table S2

Table S3

Supplementary discussion

Materials and methods

Supplementary References

Supplementary Figures

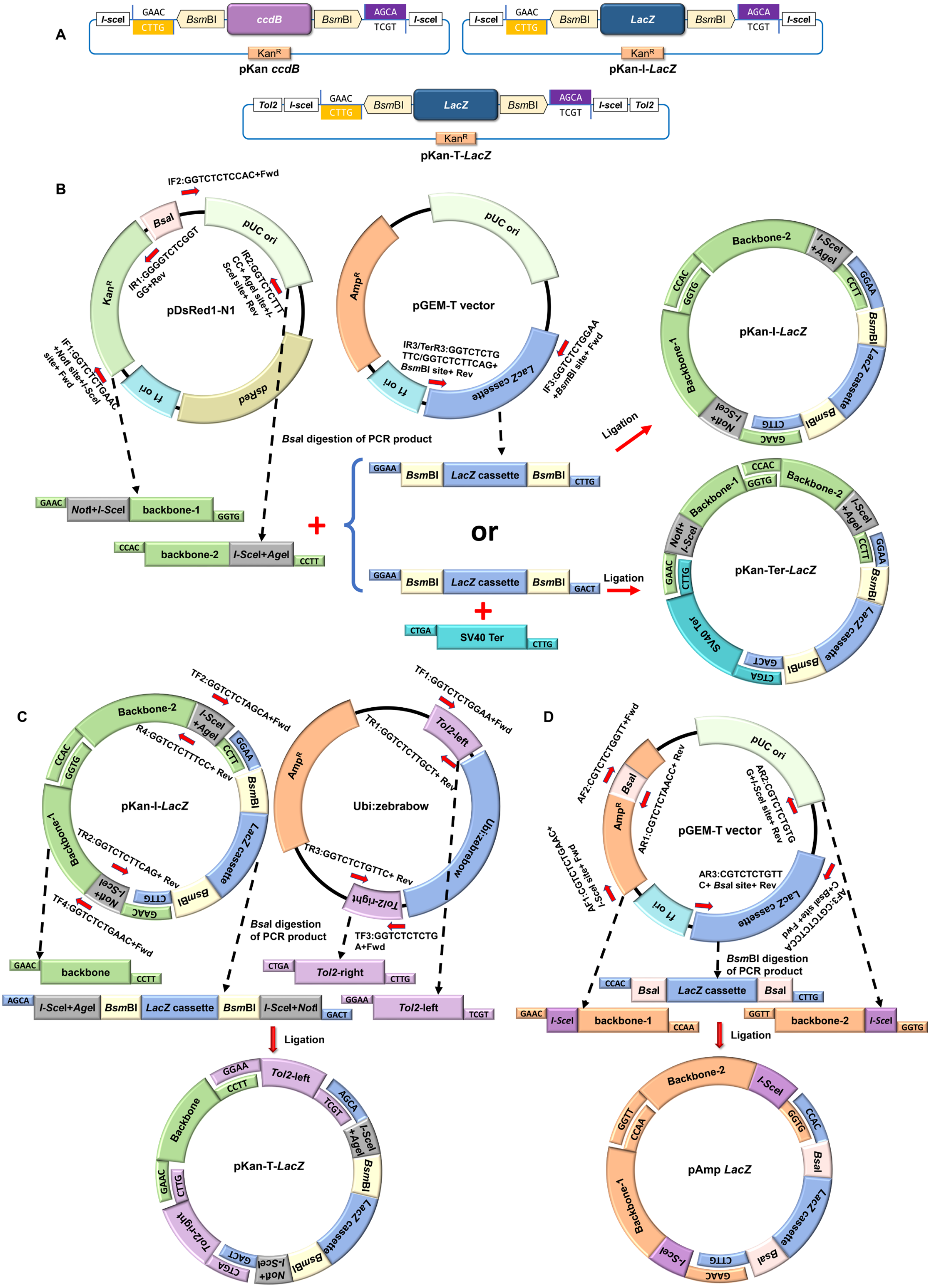

Figure S1. Recipient vectors and their generation

(A) Three recipient vectors in the GoldenFish system.

(B) Two recipient vectors, pKan-I-*LacZ* and pKan-Ter-*LacZ*, were generated by modifying the pDsRed1-N1 vector using Golden Gate cloning.

(C) Using pKan-I-*LacZ* as a template, pKan-T-*LacZ* was generated by adding *Tol2* left and right arms to the upstream and downstream of *LacZ*.

(D) The method of generating pAmp *LacZ* by modifying the pGEM-T vector.

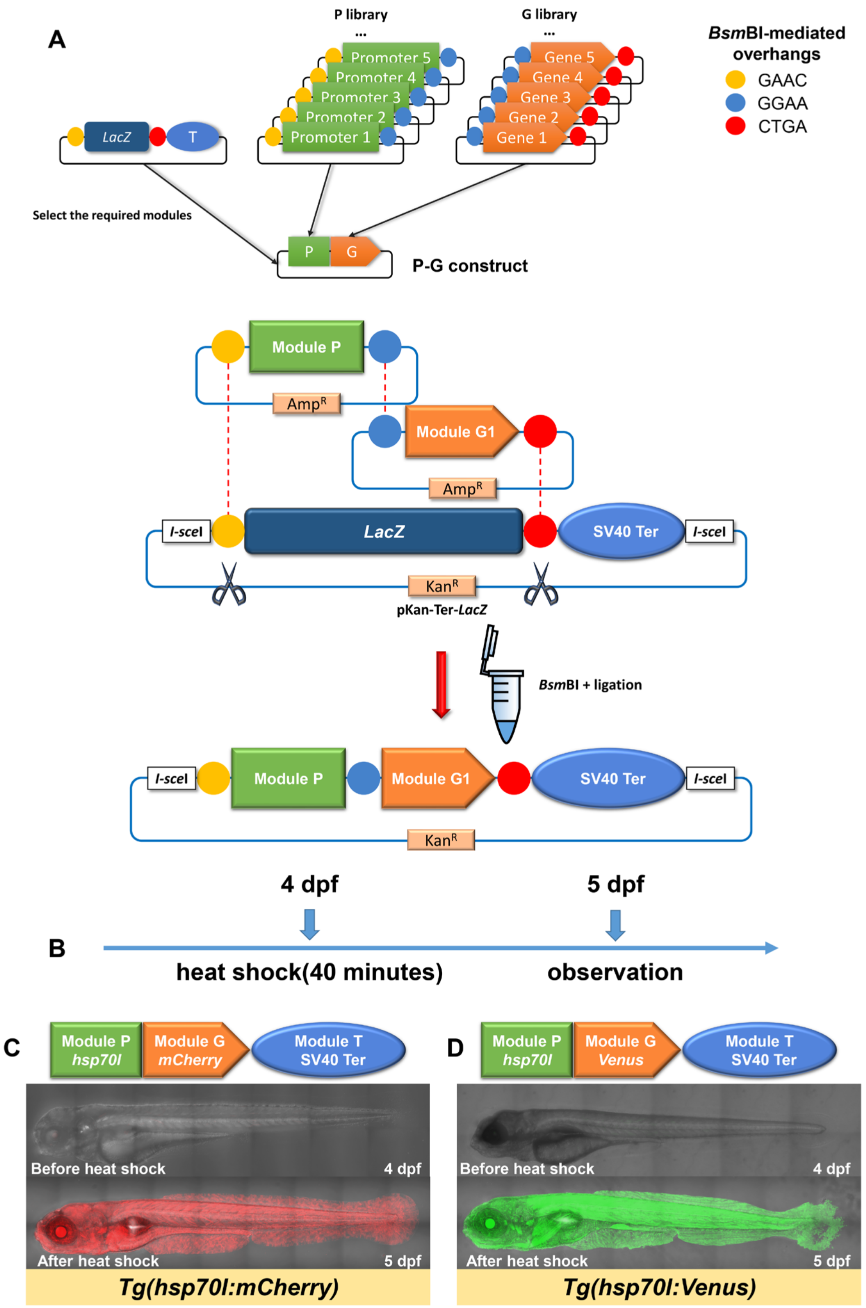

Figure S2. P-G-T construct generated by pKan-Ter-*LacZ* and basic transgenic fish generated by GoldenFish.

(A) Using the vector pKan-Ter-*LacZ* which contains the SV40 terminator downstream of *lacZ* and upstream of *I-Sce*I, only two modules are needed to generate the P-G-T construct.

(B) Heat shock strategy: heat-shocked for 40 minutes at 4 dpf (days post fertilization) and observed at 5 dpf.

(C and D) Confocal images of zebrafish injected with P-G-T constructs expressing fluorescent protein mCherry and Venus driven by heat shock promoter *hsp70l*.

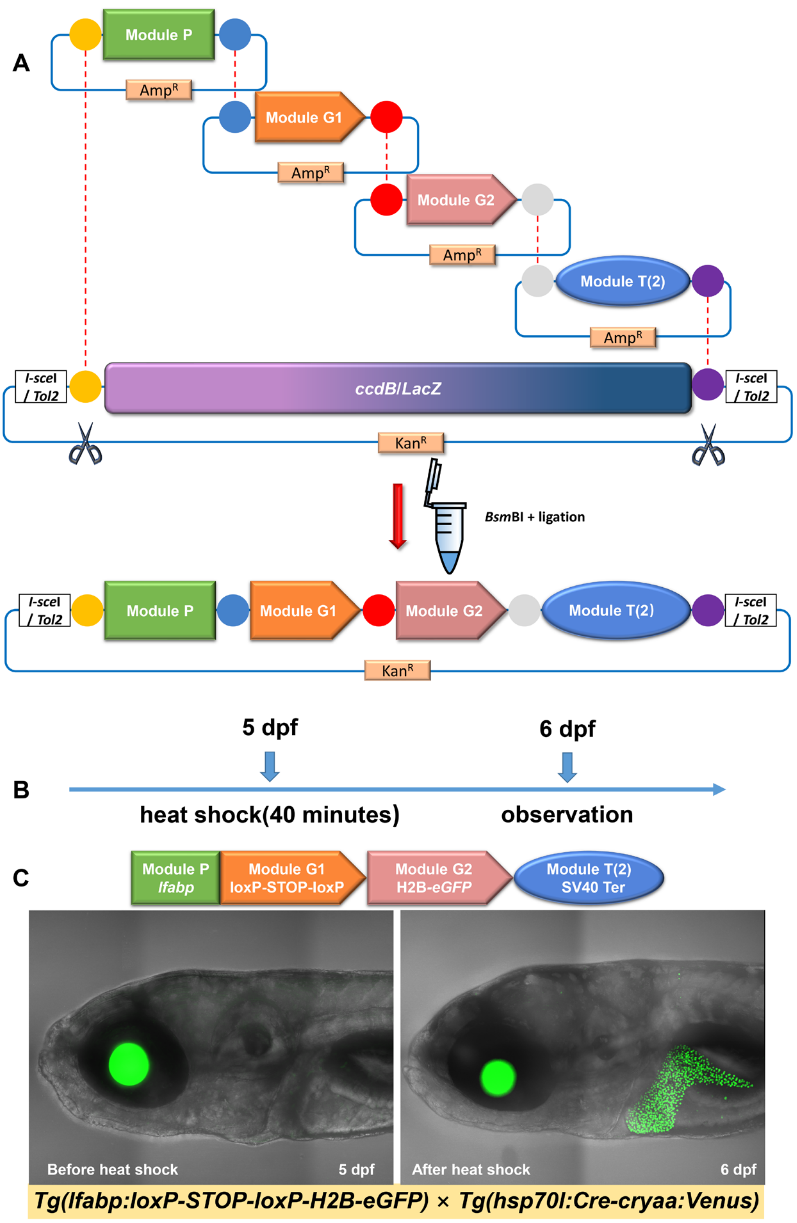

Figure S3. Overview of GoldenFish for generating P-G1.G2…Gn-T construct: multiple genes or sequences driven by one promoter.

(A) Four modules (Module P, Module G1, Module G2 and Module T(2)) flanked by *Bsm*BI, are assembled in the GoldenFish recipient vector in a predetermined direction by using the overhangs of GAAC, GGAA, CTGA, CCAC, and AGCA to complete the purpose of one promoter driving two genes.

(B) Heat shock strategy: heat-shocked for 40 minutes at 5 dpf and observed at 6 dpf.

(C) Confocal images of *Tg(lfabp:loxP-STOP-loxP-H2B-eGFP)* which identified with the background of *Tg(hsp70l:Cre-cryaa:Venus)*. After heat shock, the nucleus of the hepatocytes was labeled with eGFP fluorescence.

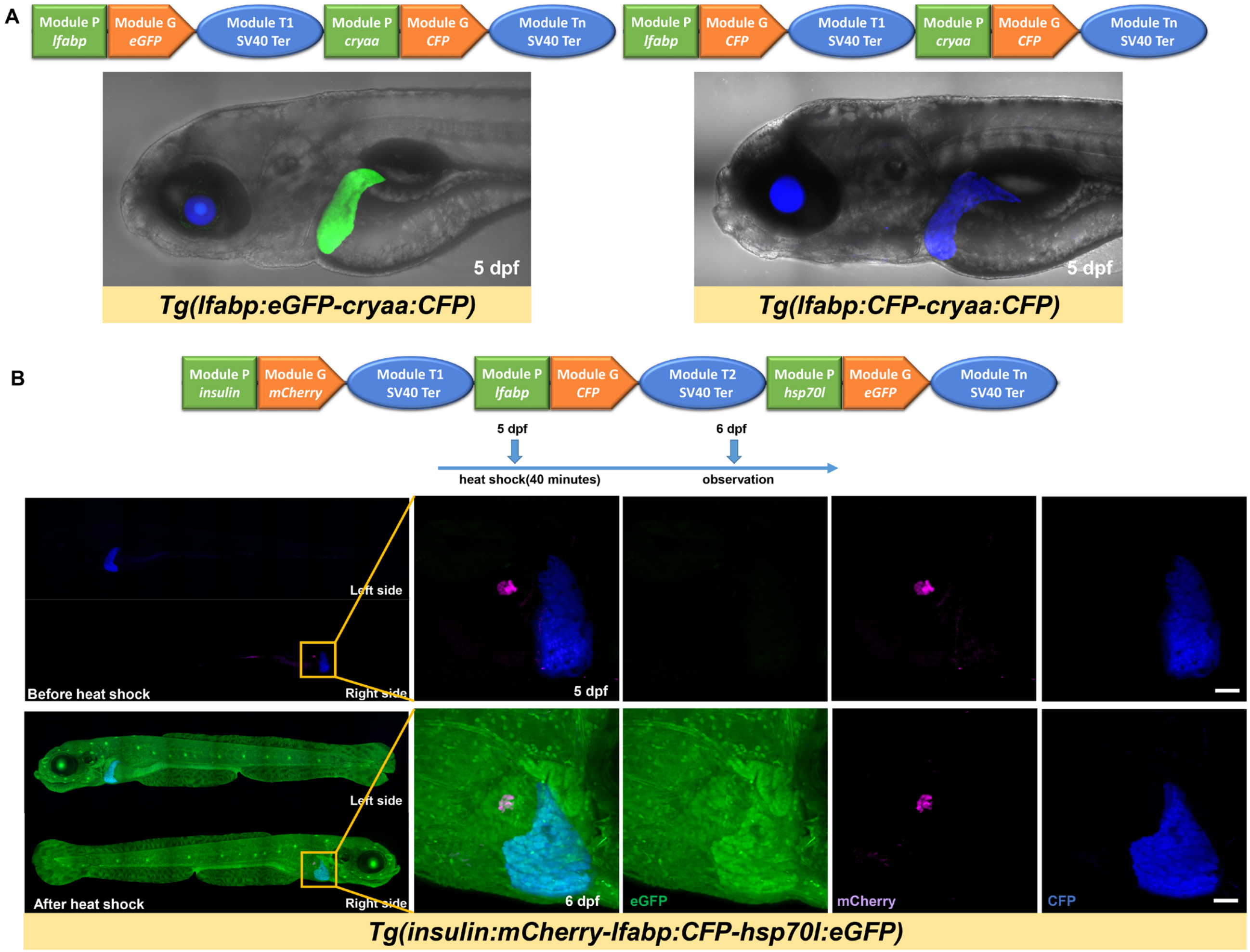

Figure S4. Multi-transgenic fish generated by GoldenFish.

(A) Lens-specific (*cryaa* drive) and liver-specific (*lfabp* drive) single TUs were first generated by a *Bsm*BI Golden Gate reaction, respectively. Then, these single TUs were assembled by a *Bsa*I Golden Gate reaction. Confocal images of double-transgenic lines express both blue fluorescent proteins in the eyes and green or blue fluorescent proteins in the liver.

(B) Confocal images of *Tg(insulin:mCherry-lfabp:CFP-hsp70l:eGFP).* After heat shock, it simultaneously expressed different colored fluorescent proteins in the pancreatic β cells, liver, and whole body. Scale bars=50 μm.

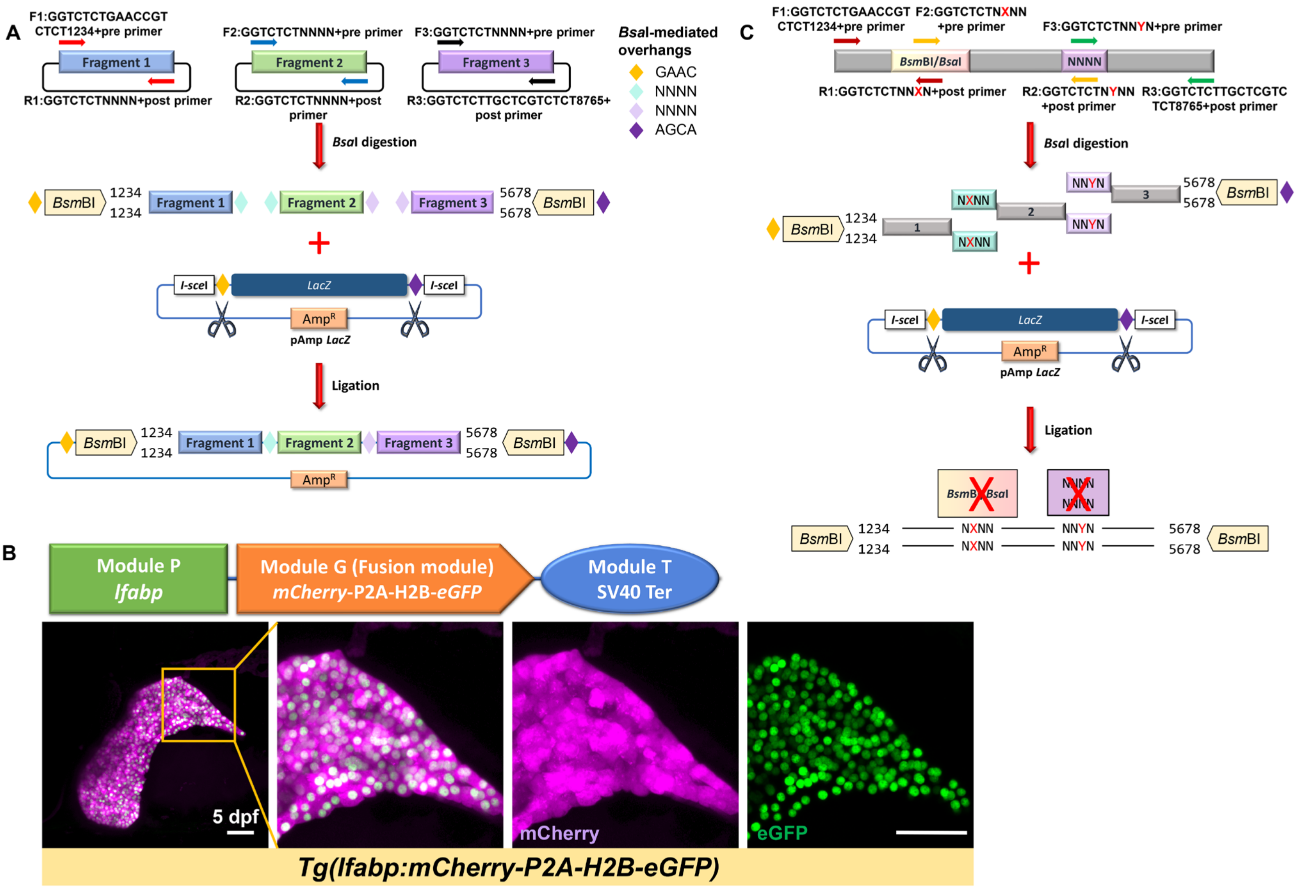

Figure S5. Generation of fusion module.

(A) Overview of the method for generating fusion modules. All fragments are amplified by PCR, adding *Bsa*I extensions to the primers. Meanwhile, *Bsm*BI sites are added upstream of the first fragment and downstream of the last one. NNNN can be any base. 1234 and 5678 represent bases, which are selected according to the library where the modules are located.

(B) Confocal images of zebrafish generated by a plasmid containing a fusion module of *mCherry*-P2A-H2B-*eGFP* driven by the liver-specific promoter. This fusion module consists of three fragments: *mCherry* with stop codon removed, H2B, and *eGFP*. Half of the linker P2A peptide with a synonymous mutation deleting the *Bsm*BI site was added to the downstream of *mCherry*, and the other half was added to the upstream of H2B by PCR. Scale bars=50 μm.

(C) Another application of the fusion module to create point mutations, including the domestication of internal illegal sites (*Bsa*I or *Bsm*BI). X and Y represent mutated bases. The primers carrying overhangs are designed where the mutations are required. Overhangs overlap mutation sites and introduce a synonymous mutation in the protein-coding sequence. In non-coding regions, it is necessary to consider whether the mutation will change the original function.

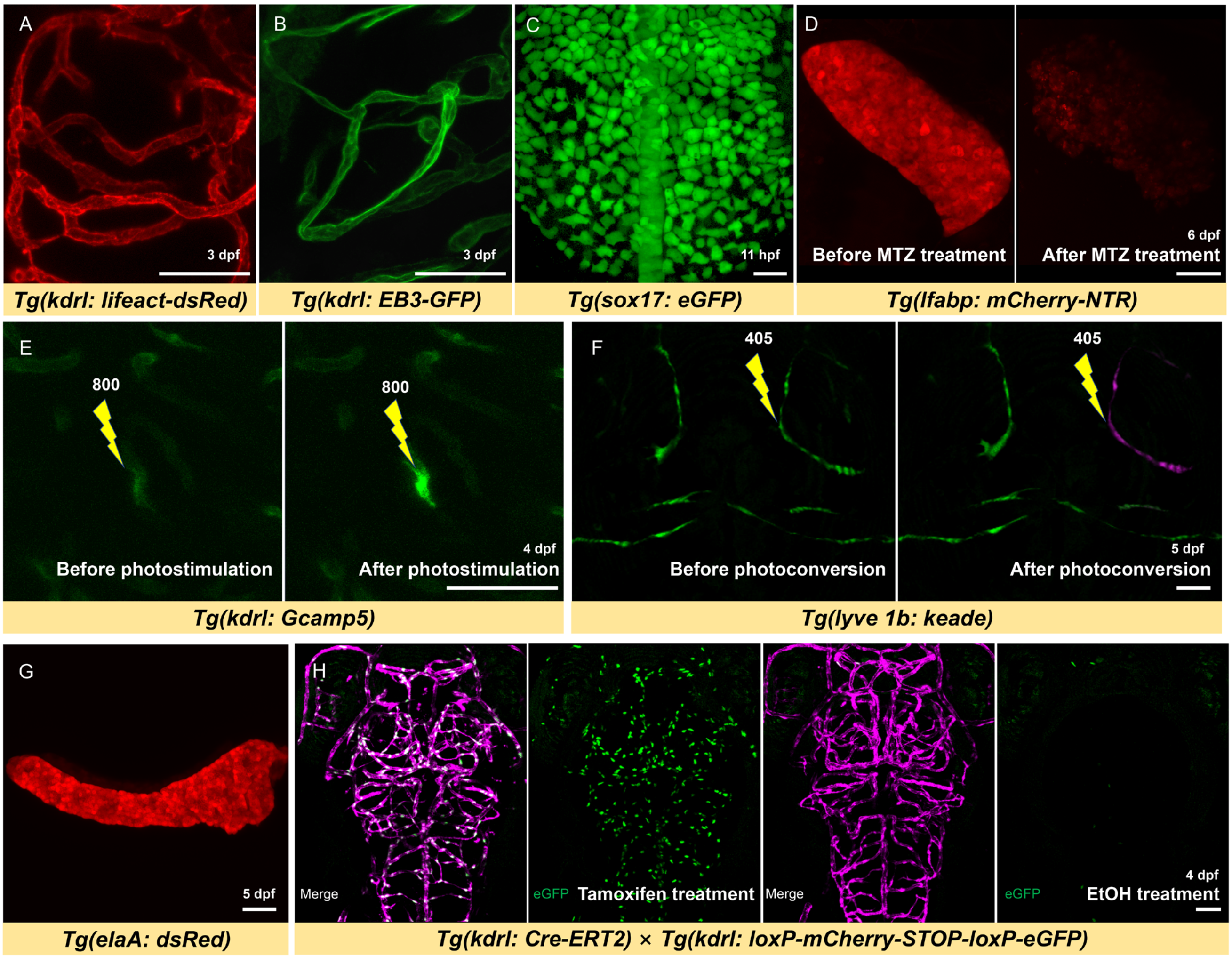

Figure S6. Additional module validation.

(A and B) Confocal images of microfilaments (lifeact-dsRed) and microtubules (EB3-GFP) driven by the vascular-specific promoter *kdrl*.

(C) Confocal images of green fluorescent protein driven by the promoter of *sox17* that marked all the early endodermal cells.

(D) Fluorescent protein mCherry fused to the bacterial nitroreductase (NTR) interacts with its substrate metroni-dazole (MTZ) to induce conditional targeted cell ablation.

(E) *Tg(kdrl: Gcamp5)* confocal image. An 800-nm laser with low energy can promote calcium ion internal flow.

(F) *Tg(lyve 1b: keade)* confocal image, expressing light conversion fluorescent protein Kaede.

(G) Confocal image of red fluorescent protein driven by the pancreatic exocrine cell-specific promoter *elaA*.

(H) CRE-ERT2 enters the nucleus under the induction of 4-Hydroxytamoxifen and excises the floxed-cassette. Scale bars=50 μm.

Supplementary Table S1. Available modules and recipient vectors

Library	Function	Name	Module type	Source	Forward primer(5'→3')	Reverse primer(5'→3')	*Bsm*BI Prefix	*Bsm*BI Suffix	*Bsa*I Prefix	*Bsa*I Suffix	Cloning methods and vectors	Bacterial resistance
**P library**	Promotor	lfabp	Basic module	(He et al., 2014)	CGTCTCTGAACCAGAATGGGGAAGGAAGAGG	CGTCTCTTTCCCTAGCGATCAACACAAAG	GAAC	GGAA	/	/	TA cloning T-vector	Ampicillin
		insulin	Basic module	(Zhong et al., 2019)	CGTCTCTGAACCGGTATCGATAAGCTTTGTTTG	CGTCTCTTTCCGGATCCGTCACACTGACACA	GAAC	GGAA	/	/	TA cloning T-vector	
		cryaa	Basic module	(Chen et al., 2019)	CGTCTCTGAACATTAATAGTGTGCATTCAGTGC	CGTCTCTTTCCGACCTGGTAACTCCTTACTG	GAAC	GGAA	/	/	TA cloning T-vector	
		elaA	Mutation module (containing a *Bsa*I site)	zebrafish genome	F1:GGTCTCTGAACCGTCTCTGAACGGTCGACGGTATCGATAAGC	R1:GGTCTCTAACCACCGGAGAGATCATGAGGTT	GAAC	/	GAAC	GGTT	Golden gate cloning pAmp *LacZ*	
					F2:GGTCTCTGGTTTCCTTTATCTTTGACAGATTTCAG	R2:GGTCTCTTGCTCGTCTCTTTCCGGATCCGCAACACTGGCATC	/	GGAA	GGTT	AGCA		
		hsp70l	Mutation module (containing two *Bsa*I sites)	addgene (Plasmid #24334)	F1:GGTCTCTGAACCGTCTCTGAACGGGATAACAGGGTAATGCGC	R1:GGTCTCTGAGTCCCCAGTGTGGTCAACAATC	GAAC	/	GAAC	ACTC		
					F2:GGTCTCTACTCATTAAGTTAGATTAAAGAC	R2:GGTCTCTGGTGTCTCTTTCGCCCTTTAAATAC	/	/	ACTC	CACC		
					F3:GGTCTCTCACCGCAGAGAAACTCAACCG	R3:GGTCTCTTGCTCGTCTCTTTCCGAGTCGACGGTATCGATCAG	/	GGAA	CACC	AGCA		
		β-actin	Mutation module (containing a *Bsa*I site)	zebrafish genome	F1:GGTCTCTGAACCGTCTCTGAACGGGTAATGCCCAAGCTGAAG	R1:GGTCTCTGGTGTCAAAGGCCGAGATAAGCGC	GAAC	/	GAAC	CACC		
					F2:GGTCTCTCACCGGGAGGGAGTGGTCGC	R2:GGTCTCTTGCTCGTCTCTTTCCGGATCTGCAGAATTCGCCCT	/	GGAA	CACC	AGTA		
		sox17	Mutation module (containing a *Bsm*BI site)	(Yang et al., 2021)	F1:GGTCTCTGAACCGTCTCTGAACTCTTCGCTATTACGCCAGCTG	R1:GGTCTCTTGTCTCTGCCCTGCATTCATC	GAAC	/	GAAC	GACA		
					F2:GGTCTCTGACAACCCCCAGGTCAACCTACAC	R2:GGTCTCTTGCTCGTCTCTTTCCGTTGCATCGACCGGTAATGC	/	GGAA	GACA	AGTA		
**G(G1) library**	Fluorescent protein	eGFP	Basic module	pEGFP-N1 (Clontech)	CGTCTCTGGAAGCCACCATGGTGAGCAAGGG	CGTCTCTTCAGTTACTTGTACAGCTCGTCCATG	GGAA	CTGA	/	/	TA cloning T-vector	Ampicillin
		mCherry	Basic module	pmCherry-N1 (Clontech)	CGTCTCTGGAAGCCACCATGGTGAGCAAGGG	CGTCTCTTCAGTTACTTGTACAGCTCGTCCATG	GGAA	CTGA	/	/	TA cloning T-vector	
		venus	Basic module	Addgene (plasmid # 54651)	CGTCTCTGGAAGCCACCATGGTGAGCAAGGG	CGTCTCTTCAGTTACTTGTACAGCTCGTCCATG	GGAA	CTGA	/	/	TA cloning T-vector	
		dsRed	Basic module	pDsRed1-N1 (Clontech)	CGTCTCTGGAAGCCACCTGGTGCGCTCCTCCAAGAA	CGTCTCTTCAGCTACAGGAACAGGTGGTGGC	GGAA	CTGA	/	/	TA cloning T-vector	
		CFP	Basic module	pECFP-N1 (Clontech)	CGTCTCTGGAAGCCACCATGGTGAGCAAGGG	CGTCTCTTCAGTTACTTGTACAGCTCGTCCATG	GGAA	CTGA	/	/	TA cloning T-vector	
		eGFP (No stop codon)	Basic module	pEGFP-N1 (Clontech)	CGTCTCTGGAAGCCACCATGGTGAGCAAGGG	CGTCTCTTCAGCTTGTACAGCTCGTCCATGC	GGAA	CTGA	/	/	TA cloning T-vector	
		mCherry (No stop codon)	Basic module	pmCherry-N1 (Clontech)	CGTCTCTGGAAGCCACCATGGTGAGCAAGGG	CGTCTCTTCAGCTTGTACAGCTCGTCCATGC	GGAA	CTGA	/	/	TA cloning T-vector	
		CFP (No stop codon)	Basic module	pECFP-N1 (Clontech)	CGTCTCTGGAAGCCACCATGGTGAGCAAGGG	CGTCTCTTCAGCTTGTACAGCTCGTCCATGC	GGAA	CTGA	/	/	TA cloning T-vector	
	Light conversion fluorescent protein	hKikGR1	Basic module	(Yang et al., 2021)	CGTCTCTGGAAGCCACCATGGTGAGCGTGATCACCA	CGTCTCTTCAGTTACTTGGCCAGCCTGGGC	GGAA	CTGA	/	/	TA cloning T-vector	
		keade	Basic module	(Chen et al., 2019)	CGTCTCTGGAAGCCACCATGAGTCTGATTAAACCAGAA	CGTCTCTTCAGTTACTTGACGTTGTCCGGCA	GGAA	CTGA	/	/	TA cloning T-vector	
		dendra2	Basic module	(He et al., 2014)	CGTCTCTGGAAGCCACCATGAACACTCCTGG	CGTCTCTTCAGTTAGTACACACCTGAGTCTC	GGAA	CTGA	/	/	TA cloning T-vector	
	Nitroreductase to convert MTZ into a cytotoxic metabolite	mCherry-NTR	Basic module	(He et al., 2014)	CGTCTCTGGAAGCCACCATGGTGAGCAAGGG	CGTCTCTTCAGTTACACTTCGGTTAAGGTGATG	GGAA	CTGA	/	/	TA cloning T-vector	
		CFP-NTR	Basic module	(Chen et al., 2019)	CGTCTCTGGAAGCCACCATGGTGAGCAAGGG	CGTCTCTTCAGTTACACTTCGGTTAAGGTGATG	GGAA	CTGA	/	/	TA cloning T-vector	
		dendra2-NTR	Basic module	(He et al., 2014)	CGTCTCTGGAAGCCACCATGAACACTCCTGG	CGTCTCTTCAGTTACACTTCGGTTAAGGTGATG	GGAA	CTGA	/	/	TA cloning T-vector	
	GAL4/UAS System element	gal4-FF	Mutation module (containing a *Bsa*I site)	pT2KSAGFF (provided by Kawakami)	F1:GGTCTCTGAACCGTCTCTGGAATGAAGCTACTGTCTTCTATC	R1:GGTCTCTTCTTCTTTTGGTTTTGGGAGAGTAG	GGAA	/	GAAC	AAGA	TA cloning T-vector	
					F2:GGTCTCTAAGATCTCCGCTGACTAGGGCACA	R2:GGTCTCTTGCTCGTCTCTTCAGTTAGTTACCCGGGAGCATATCG	/	CTGA	AAGA	AGCA		
	Lineage-tracing element	loxP-STOP-loxP	Basic module	(Chen et al., 2021)	CGTCTCTGGAAGATCAGCTTGGGCTGCAGGT	CGTCTCTTCAGGAGGGGATCGATCCGGAACC	GGAA	CTGA	/	/	TA cloning T-vector	
		loxP-mcherry-STOP-loxP	Basic module	addgene (Plasmid #24334)	CGTCTCTGGAAGATCAGCTTGGGCTGCAGGT	CGTCTCTTCAGGCCACCGGTGGATCCGTCGA	GGAA	CTGA	/	/	TA cloning T-vector	
		Cre	Mutation module (containing a *Bsm*BI site)	addgene (Plasmid #105537)	F1:GGTCTCTGAACCGTCTCTGGAAGCCACCATGTCCAATTTACTGACCGTAC	R1:GGTCTCTGACTGAAATCCATCGCTCGACCAG	GGAA	/	GAAC	AGTC	Golden gate cloning pAmp *LacZ*	
					F2:GGTCTCTAGTCTCTGGTGTAGCTGATGA	R2:GGTCTCTTGCTCGTCTCTTCAGCTAATCGCCATCTTCCAGCAG	/	CTGA	AGTC	AGCA		
		Cre-ERT2	Mutation module (containing a *Bsm*BI site and two *Bsa*I sites)	addgene (Plasmid #62427)	F1:GGTCTCTGAACCGTCTCTGGAAGCCACCATGTCCAATTTACTG	R1:GGTCTCTGACTGAAATCCATCGCTCGACCAG	GGAA	/	GAAC	AGTC		
					F2:GGTCTCTAGTCTCTGGTGTAGCTGATGA	R2:GGTCTCTGTCCAATCATCAGGATCTCTAG	/	/	AGTC	GGAC		
					F3:GGTCTCTGGACTCGTCTGGCGCTCCATGG	R3:GGTCTCTCAGTCTCCTCCACGGATGCCC	/	/	GGAC	ACTG		
					F4:GGTCTCTACTGACCAAAGCCACTTGGCCAC	R4:GGTCTCTTGCTCGTCTCTTCAGTCAAGCTGTGGCAGGGAAAC	/	CTGA	ACTG	AGCA		
	Labeled microtubules	EB3-GFP	Basic module	addgene (Plasmid #105948)	CGTCTCTGGAAGCCACCATGGCCGTCAATG	CGTCTCTTCAGTTACTTGTACAGCTCGTCCA	GGAA	CTGA	/	/	TA cloning T-vector	
	Labeled microfilaments	lifeact-dsRed	Mutation module (containing a *Bsa*I site)	(Liu et al., 2016)	F1:GGTCTCTGAACCGTCTCTGGAAGCCACCATGGGTGTCGCAGATTTGATC	R1:GGTCTCTGAGTCTCGCCCTTCAGCACGC	GGAA	/	GAAC	ACGC	Golden gate cloning pAmp *LacZ*	
					F2:GGTCTCTACTCACAAGGCCCTGAAGCTGAA	R2:GGTCTCTTGCTCGTCTCTTCAGTTACAGGAACAGGTGGTGGC	/	CTGA	ACGC	AGCA		
	Mark changes in calcium concentration	Gcamp5	Mutation module (containing a *Bsa*I site)	addgene (Plasmid #31788)	F1:GGTCTCTGAACCGTCTCTGGAACGCCACCATGGGTTCTCATC	R1:GGTCTCTATCGCATCACCGTCCCCAGCTC	GGAA	/	GAAC	CGAT	Golden gate cloning pAmp *LacZ*	
					F2:GGTCTCTCGATCTCTGGGGCAGAACCCCAC	R2:GGTCTCTTGCTCGTCTCTTCAGTCACTTCGCTGTCATCATTTG	/	CTGA	CGAT	AGCA		
	Fluorescent protein+linker+fluorescent protein	mCherry-P2A-H2B-eGFP	Fusion module	addgene (Plasmid #24334)	F1:GGTCTCTGAACCGTCTCTGGAAGCCACCATGAACACTCCTGGAATCAATC	R1:GGTCTCTGCTTCAGCAGGCTGAAGTTAGTAGCTCCGCTTCCCTTGTACAGCTCGTCCATGC	GGAA	/	GAAC	AAGC	Golden gate cloning pAmp *LacZ*	
					F2:GGTCTCTAAGCAGGCTGGAGATGTGGAGGAGAACCCTGGACCTATGCCAGAGCCAGCGAAGTC	R2:GGTCTCTCTTAGCGCTGGTGTACTTGG	/	/	AAGC	TAAG		
					F3:GGTCTCTTAAGATGGTGAGCAAGGGCG	R3:GGTCTCTTGCTCGTCTCTTCAGTTACTTGTACAGCTCGTCCATG	/	CTGA	TAAG	AGCA		
**G2 library**	Fluorescent protein	eGFP	Basic module	pEGFP-N1 (Clontech)	CGTCTCTCTGATGGTGAGCAAGGGCGAGGA	CGTCTCTGTGGTTACTTGTACAGCTCGTCCATG	CTGA	CCAC	/	/	TA cloning T-vector	Ampicillin
		H2B-eGFP	Basic module	addgene (Plasmid #24334)	CGTCTCTCTGAATGCCAGAGCCAGCGAAGTC	CGTCTCTGTGGTTACTTGTACAGCTCGTCCATG	CTGA	CCAC	/	/	TA cloning T-vector	
		mCherry	Basic module	pmCherry-N1 (Clontech)	CGTCTCTCTGATGGTGAGCAAGGGCGAGGA	CGTCTCTGTGGTTACTTGTACAGCTCGTCCATG	CTGA	CCAC	/	/	TA cloning T-vector	
		venus	Basic module	Addgene (plasmid # 54651)	CGTCTCTCTGATGGTGAGCAAGGGCGAGGA	CGTCTCTGTGGTTACTTGTACAGCTCGTCCATG	CTGA	CCAC	/	/	TA cloning T-vector	
		CFP	Basic module	pECFP-N1 (Clontech)	CGTCTCTCTGATGGTGAGCAAGGGCGAGGA	CGTCTCTGTGGTTACTTGTACAGCTCGTCCATG	CTGA	CCAC	/	/	TA cloning T-vector	
		dsRed	Basic module	pDsRed1-N1 (Clontech)	CGTCTCTCTGATGGTGCGCTCCTCCAAGAA	CGTCTCTGTGGCTACAGGAACAGGTGGTGGC	CTGA	CCAC	/	/	TA cloning T-vector	
**T library**	Terminator (SV40 PolyA terminator)	T1	Basic module	pECFP-N1 (Clontech)	CGTCTCTCTGACCATACCACATTTGTAGAGG	CGTCTCTTGCTCGCCTTAAGATACATTGATGAG	CTGA	AGCA	/	/	TA cloning T-vector	Ampicillin
		T2	Basic module		CGTCTCTCCACCATACCACATTTGTAGAGG	CGTCTCTTGCTCGCCTTAAGATACATTGATGAG	CCAC	AGCA	/	/		
		T-1	Basic module		CGTCTCTCTGACCATACCACATTTGTAGAGG	CGTCTCTTGCTGGTCTCTTTCCGCCTTAAGATACATTGATGAG	CTGA	AGCA	/	GGAA		
		T-2	Basic module		CGTCTCTCTGACCATACCACATTTGTAGAGG	CGTCTCTTGCTGGTCTCTTCAGCGCCTTAAGATACATTGATGAG	CTGA	AGCA	/	CTGA		
		T-3	Basic module		CGTCTCTCTGACCATACCACATTTGTAGAGG	CGTCTCTTGCTGGTCTCTGTGGCGCCTTAAGATACATTGATGAG	CTGA	AGCA	/	CCAC		
		T-N	Basic module		CGTCTCTCTGACCATACCACATTTGTAGAGG	CGTCTCTTGCTGGTCTCTTGCTCGCCTTAAGATACATTGATGAG	CTGA	AGCA	/	AGCA		
**V library**	Recipient vector	pKan *ccdB*	/	pGl3-CMV-NLS-TAL-*ccdB*-Fok1 (Wensheng Wei lab/Peking University)/ pGEM-T Easy Vector (Promega)/ pDsRed1-N1 (Clontech)/(Pan et al., 2013)	/	/	GAAC	AGCA	/	/	/	Kanamycin
		pKan-I-*LacZ*						AGCA	/	/		
		pKan-T-*LacZ*						AGCA	/	/		
		pKan-Ter-*LacZ*						CTGA	/	/		
		pKan *ccdB*-1						AGCA	GAAC	/		
		pKan *ccdB*-2						AGCA	GGAA	/		
		pKan *ccdB*-3						AGCA	CTGA	/		
		pKan *ccdB*-4						AGCA	CCAC	/		
		pAmp *LacZ*							/	/		Ampicillin

In red: *Bsa*I site. In yellow: *Bsm*BI site. In coloured italics: *Bsm*BI or *Bsa*I recognition site. Underlined: *Bsm*BI or *Bsa*I cutting site (overhangs).

Supplementary Table S2. Expression efficiency of each transgenic line

Transgenic line	Positive expression rate in F0	Germline transmission rate in F1
Tg(hsp70l:mCherry)^{cq111}	70%-75%	25% - 35%
Tg(hsp70l:Venus)^{cq112}	70%-75%	25% - 35%
Tg(β-actin:hKikGR1)^{cq113}	75%-85%	25% - 35%
Tg(lfabp:loxP-STOP-loxP-H2B-eGFP)^{cq114}	70%-75%	40%-45%
Tg(lfabp:loxP-mCherry-STOP-loxP-eGFP)^{cq116}	70%-75%	35%-45%
Tg(lfabp:GFP-cryaa:CFP)^{cq117}	55%-60%	35%-40%
Tg(lfabp:CFP-cryaa:CFP)^{cq118}	50%-60%	35%-40%
Tg(insulin:mCherry-lfabp:CFP-β-actin:Venus)^{cq119}	40%-50%	20%-30%
Tg(insulin:mCherry-lfabp:CFP-hsp70l:eGFP)^{cq120}	40%-50%	20%-30%
Tg(lfabp:mCherry-P2A-H2B-eGFP)^{cq121}	70%-75%	25%-30%

The statistics among multi-transgenic fish were the condition that all genes were simultaneously expressed.

Supplementary Table S3. Generation of recipient vectors used in this paper

Name	Element	Template	Forward primer(5'→3')	Reverse primer(5'→3')	Construction method	Cloning vectors
pKan *ccdB*	*ccdB* cassette	pGl3-CMV-NLS-TAL-*ccdB*-Fok1	ACCGGTCTGAACAGAGACGCTGGCTTATCGAATTAATAC	TGCTAGAGACGTTCGAACCGCGGGCCCTCTA	Subcloning with *Age*I/*Not*I	pKan-I-*LacZ*
pKan-I-*LacZ*	Kana1	pDsRed1-N1	IF1:GGTCTCTGAACGCGGCCGCTAGGGATAACAGGGTAATCTTAATGCGCCGCTACAGGG	IR1: GGGGTCTCGGTGGGGTATC	Golden gate cloning	/
	Kana2	pDsRed1-N1	IF2:GGTCTCTCCACGCGTCAGACCCCGTAGAAAA	IR2:GGTCTCTTTCCACCGGTATTACCCTGTTATCCCTATGCATGGCGGTAATACGGTT		
	LacZ	pGEM-T vector	IF3:GGTCTCTGGAAGAACAGAGACGCCGAAAAGTGCCACCTGATG	IR3:GGTCTCTGTTCTGCTAGAGACGTACCGCCTTTGAGTGAGCTG		
pKan-T-*LacZ*	*Tol2* left arm	ubi:zebrabow	TF1:GGTCTCTGGAAGGTTTGTCCAGGAGTTCTTG	TR1:GGTCTCTTGCTGCGTACGAATGGCCCATCTG	Golden gate cloning	/
	LacZ+*I-Sce*I	pKan-I-*LacZ*	TF2:GGTCTCTAGCAGTTATCCCCTGATTCTGTGG	TR2:GGTCTCTTCAGAAAAGTGCCACCTGACGCG		
	Tol2 right arm	ubi:zebrabow	TF3:GGTCTCTCTGACGCGTGATCTGCGAAGATAC	TR3:GGTCTCTGTTCGAACTAGTGGATCTGCTGGG		
	Kana	pKan-I-*LacZ*	TF4:GGTCTCTGAACGCGTCAGGTGGCACTTTTC	TR4:GGTCTCTTTCCACAGAATCAGGGGATAAC		
pKan-Ter-*LacZ*	Kana1	pDsRed1-N1	IF1:GGTCTCTGAACGCGGCCGCTAGGGATAACAGGGTAATCTTAATGCGCCGCTACAGGG	IR1:GGGGTCTCGGTGGGGTATC	Golden gate cloning	/
	Kana2	pDsRed1-N1	IF2:GGTCTCTCCACGCGTCAGACCCCGTAGAAAA	IR2:GGTCTCTTTCCACCGGTATTACCCTGTTATCCCTATGCATGGCGGTAATACGGTT		
	LacZ	pGEM-T vector	IF3:GGTCTCTGGAAGAACAGAGACGCCGAAAAGTGCCACCTGATG	TerR3:GGTCTCTTCAGTCAGAGAGACGTACCGCCTTTGAGTGAGCTG		
	SV40 polyA	pEGFP-N1	TerF4:GGTCTCTCTGAATACCACATTTGTAGAGG	TerR4:GGTCTCTGTTCCGCCTTAAGATACATTGATGAG		
pAmp *LacZ*	Amp1	pGEM-T vector	AF1:CGTCTCTGAACTAGGGATAACAGGGTAATAGCATCAGGTGGCACTTTTC	AR1:CGTCTCTAACCACGCTCACCGGCTCCAG	Golden gate cloning	/
	Amp2		AF2:CGTCTCTGGTTCTCGCGGTATCATTGCAGC	AR2:CGTCTCTGTGGATTACCCTGTTATCCCTACAGCTCACTCAAAGGCGGTA		
	LacZ		AF3:CGTCTCTCCACTGCTAGAGACCTACCGCCTTTGAGTGAGCTG	AR3:CGTCTCTGTTCGAACAGAGACCGAAAAGTGCCACCTGATG		
pKan *ccdB*-1	*ccdB* cassette	pKan *ccdB*	ACCGGTGGTCTCTGAACGAACAGAGACGCTGGCTTATC	TGCTAGAGACGTTCGAACCG	Subcloning with *Age*I/*Not*I	pKan-I-*LacZ*
pKan *ccdB*-2	*ccdB* cassette		ACCGGTGGTCTCTGGAAGAACAGAGACGCTGGCTTATC	TGCTAGAGACGTTCGAACCG		
pKan *ccdB*-3	*ccdB* cassette		ACCGGTGGTCTCTCTGAGAACAGAGACGCTGGCTTATC	TGCTAGAGACGTTCGAACCG		
pKan *ccdB*-4	*ccdB* cassette		ACCGGTGGTCTCTCCACGAACAGAGACGCTGGCTTATC	TGCTAGAGACGTTCGAACCG		

In red: *Bsa*I site. In yellow: *Bsm*BI site. In coloured italics: *Bsm*BI or *Bsa*I recognition site. Underlined: *Bsm*BI or *Bsa*I cutting site (overhangs). In blue: AgeI site. In purple: NotI site. In black bold: I-sceI site.

Supplementary discussion

Traditionally, generating constructs for transgenesis mainly relies on subcloning sequences of interest into the existing expression plasmid through the use of type II restriction enzymes to produce complementary ends and DNA Ligase to seal the integration (Cohen et al., 1973; Roberts, 2005), which requires suitable restriction sites and existing plasmids that can be modified. Moreover, it typically connects only two DNA parts at a time. For a more complex construct, researchers must design various cloning steps needing different restriction enzymes. Therefore, more simplified and convenient technologies such as Gateway cloning (Hartley et al., 2000), In-Fusion cloning (Sleight et al., 2010), Gibson Assembly (Gibson et al., 2009), and USER cloning (Geu-Flores et al., 2007) have emerged. They all have their advantages and characteristics. For recombination-based Gateway cloning, specific recombination sites will remain in the final construct and cannot be removed, which will affect gene expression or protein function. The other three methods need linearized fragments to introduce overlapping ends and cannot be long-saved for subsequent use. Moreover, In-Fusion cloning and Gibson assembly required a 15-40 bp long overlapping region at both ends of the ligating fragment. If multiple homologous genes (such as different fluorescent proteins) are connected, they cannot work because their long overlapping regions are the same or similar. These characteristics prevent these approaches from forming a systematic solution to construct generation.

"Golden Gate" cloning is a widely used DNA assembly method, that first appeared in 2008 (Engler et al., 2008; Engler et al., 2009). Since then, it has been applied to many fields, such as designing transcriptional activator-like effectors (TALEs) (Weber et al., 2011), plant Synthetic Biology (Patron et al., 2015), yeast (Mitchell et al., 2015), and mammalian cells (Fonseca et al., 2019). They are flexible, efficient, and easy to operate. To establish a one-step, efficient, and systematic method to generate constructs for transgenesis, we take zebrafish transgenesis as an example and develop a GoldenFish system, which makes up for the gap in this aspect. It covers almost all types of transgenic fish and makes it possible to assemble complex DNA constructs.

To simplify our system and minimize the number of connected modules, we only designed 3-4 necessary libraries, which can achieve 95%-100% of the connection efficiency and meet the needs of most transgenic fish. For a small number of complex transgenic constructs, the need for increased tags or genes, etc., can be achieved by fusion module formats or adding additional modules. Meanwhile, we provide two most commonly used transgenic methods for selection, *I-Sce*I and *Tol2*.

The "Lego block" modules bring the advantages that the construct can be customized. The addition of the new module is very simple, just needs to amplify the required sequence according to the rules, and clone the PCR product into T-vector. As the number of constructions increases, modules in the library are becoming increasingly enriched over time. Once the library is generated, the modules stored in the same library can be quickly replaced for different constructs, and only one or two reactions can complete the generation.

To validate our system, various types of plasmids, simple and complex, have been generated and successfully expressed in zebrafish. All the fish line images shown in the main text are F2 or F3 generations. Until now, they have reached at least F3 generation or later, which means our system can obtain stable transgenic lines. From the experimental results, the rate of transgenesis efficiency is high (Supplementary Table S2). Especially for multi-transgenic fish, the traditional method is to generate single-transgenic fish respectively and then cross them together. It has several disadvantages: Firstly, it needs to be injected and screened separately. Secondly, multiple rounds of screening and hybridization will take a lot of time and manpower. Thirdly, in the screening process of hybrid generation, there will be complex character segregation that is unwilling to appear. Our method can overcome these deficiencies and easily generate the construct containing multiple transgenes at one time, which shortens the work to three months to obtain stable transgenic fish lines.

To enrich the components of the libraries, in addition to the components listed in the main text, we made some supplements as the initially created libraries. The generation method is shown in Table S1. Because it is a start-up, the number of components is limited, but it can be gradually enriched in subsequent use, providing convenience for future users. After verification, all start-up components can work well (Supplementary Figure S6).

We use constructs for zebrafish transgenesis as an example. By designing appropriate recipient vectors, our system can also be applied to other transgenic organisms and these existing modules can be used across species, which can greatly improve the universality of our system.

Materials and methods

Ethics statement

All experimental protocols were approved by the School of Life Sciences, Southwest University (Chongqing, China), and the methods were carried out following the approved guidelines. The use of animals was in accordance with the regulations of the ethics committee of Southwest University (Chongqing, China). Zebrafish were maintained following the Guidelines of Experimental Animal Welfare from the Ministry of Science and Technology of the People's Republic of China (2006) and the Institutional Animal Care and Use Committee protocols from Southwest University (2007).

Zebrafish strains

All zebrafish lines *Tg(hsp70l:mCherry)*^{cq111}, *Tg(hsp70l:Venus)*^{cq112}, *Tg(β-actin:hKikGR1)*^{cq113}, *Tg(lfabp:loxP-STOP-loxP-H2B-eGFP)*^{cq114}, *Tg(hsp70l:Cre; cryaa:Venus)*^{cq115}, *Tg(lfabp:loxP-mCherry-STOP-loxP-eGFP)*^{cq116}, *Tg(lfabp:GFP; cryaa:CFP)*^{cq117}, *Tg(lfabp:CFP; cryaa:CFP)*^{cq118}, *Tg(insulin:mCherry-lfabp:CFP-β-actin:Venus)*^{cq119} *Tg(insulin:mCherry-lfabp:CFP-hsp70l:eGFP)*^{cq120}, *Tg(lfabp:mCherry-P2A-H2B-eGFP)*^{cq121} were raised and maintained under standard laboratory conditions according to Institutional Animal Care and Use Committee protocols. To facilitate photographing, embryos were cultured in 0.003% 1-phenyl-2-thiourea (PTU, Sigma) to inhibit pigment production.

Recipient vectors generation

The Recipient vector pKan-I-*LacZ*, pKan-T-*LacZ*, pKan-Ter-*LacZ*, and pAmp *LacZ* were generated by connecting three or four components through Golden gate cloning, while pKan *ccdB* and pKan *ccdB*-1 to 4 were generated by traditional subcloning methods. Strategies and primers are summarized in Table S3.

The vector pKan-I-*LacZ* was modified from pDsRed1-N1 which contained an unwanted *Bsa*I site located in a non-essential region (Supplementary Figure S1B). To remove this site, we designed IR1 upstream and IF2 downstream of this site. At the same time, IF1 carrying *Not*I and *I-Sce*I sites were designed downstream of f1 *ori* which is an unnecessary element in our vector, and IR2 carrying *Age*I and *I-Sce*I sites were designed downstream of the pUC origin of replication. In this way, the backbone was divided into two fragments, backbone-1, and backbone-2. The *lacZ* cassette, derived from the pGEM-T vector, was designed flanked by two *Bsm*BI sites that produce overhangs GAAC and AGCA which are compatible with overhangs from Promoters and Terminators via IF3 and IR3. First, we amplified these three components by PCR with the *Bsa*I site added in front of the specific forward primer sequences and obtained the PCR products flanked by *Bsa*I sites. Then, the three components were connected end to end into a ring to form pKan-I-*LacZ* by a *Bsa*I Golden Gate reaction. For pKan-Ter-*LacZ*, we only needed to add SV40 PolyA after the *lacZ* cassette and changed the corresponding overhangs to make them can be connected (Supplementary Figure S1B). For pKan-T-*LacZ*, the backbone was amplified from pKan-I-*LacZ* which the *Bsa*I site had been removed (Supplementary

Figure S1C). And the *lacZ* cassette was also from pKan-I-*LacZ* for the necessary sites such as *I-Sce*I, *Bsm*BI, *Age*I and *Not*I had already existed. Two mini*Tol2* sequences were from plasmid Ubi:zebrebow (Pan et al., 2013). After the *Bsa*I Golden Gate reaction, *Tol2* left arm was located upstream and the right arm downstream of the *lacZ* cassette to ensure that the final constructs can work smoothly through the transposase system.

For pAmp *LacZ*, all three components were derived from the pGEM-T vector (Supplementary Figure S1D). Since the coding sequence of ampicillin resistance contained a *Bsa*I site that needs to be removed, we designed two primers, AR1 and AF2, which overlapped this site and introduced a synonymous mutation to maintain the open reading frame, and then the *Bsa*I recognition sequence GGTCTC was changed into GGTTTC. At the same time, two *I-Sce*I sites were introduced to flank the *lacZ* cassette via AF1 and AR2, while the insertion of *Bsa*I sites was via AF3 and AR3. These three components were connected into a ring by a *Bsm*bI Golden Gate reaction.

For the generation of the recipient vectors pKan *ccdB*, a *ccdB* cassette was amplified using primers containing *Age*I and *Bsm*BI sites, and the PCR product was cloned into the pGEM-T easy vector (Promega, Madison, WI) without any mutagenesis. Therefore, the upstream of the *ccdB* cassette obtained the *Age*I site from the forward primer, and the downstream obtained the *Not*I site from the T-vector. Then, the *ccdB* cassette was inserted into pKan-I-*LacZ*, which had been generated previously, to generate pKan *ccdB* by replacing the *LacZ* cassette via *Age*I/*Not*I digestion. The generation method of vector pKan *ccdB*-1 to 4 was almost the same as that of pKan *ccdB*, except that the *ccdB* cassette was obtained using pKan *ccdB* as a template, and the *Bsa*I sites with different cutting sites were introduced through the forward primer.

Modules generation

It is easy to add new modules to the corresponding libraries. All modules were amplified by PCR using high-fidelity PrimeSTAR HS DNA Polymerase (Takara) or La Taq DNA polymerase (Takara) following the manufacturer's instructions. A Kozak sequence (GCCACC) (Kozak, 1987) can be added to the 5' end of the target gene by primers. The primers and methods are provided in Table S1.

Basic modules generation

The basic module was amplified by primers added with a 5' extension containing a *Bsm*BI restriction site. PCR products were cleaned up with the QIAquick Gel Extraction Kit (Qiagen). Purified PCR products consisting of the module flanked by *Bsm*BI sites were TA Cloned into pGEM-T Easy Vector (Promega) and 1 μL of the ligation was transformed into TOP10 electrocompetent cells. Positive clones were selected in ampicillin-containing plates and confirmed by Colony PCR and sequencing. Plasmid DNA was extracted using AxyPrep™ Plasmid Miniprep Kit (Axygen).

Fusion modules generation

All fragments are amplified by PCR with *Bsa*I sites added in front of the primer, and *Bsm*BI sites

whose overhangs are selected according to the library where the modules are located are added upstream of the first fragment and downstream of the last one. For the base mutation in the module, overhangs produced by *Bsa*I sites overlapped the mutation site and introduced a synonymous mutation to correct the wrong base. Then a *Bsa*I Golden Gate reaction is performed with pAmp *LacZ* as the recipient vector.

Optimization of Golden Gate reaction

All Golden Gate reactions were setup using 150 ng each of the modules (pre-cloned modules or PCR products) mixed with 150 ng recipient vector, 1 μL of the Type IIS restriction enzyme using *Bsm*BI or *Bsa*I (New England Biolabs), 1 μL of T4 DNA Ligase (Takara Biosciences), 2 μL of 10x T4 DNA Ligase buffer (Takara Biosciences) adding additional 0.1 μL ATP (25mM) and 0.2 μL DTT (100mM, Promega P117A), and the final reaction volume was adjusted to 20 μL using sterile ultrapure water.

The mixture was performed for 15-18 cycles of 37°C for 5 min and 16°C for 10 min each, followed by 37°C for 15 min and 80°C for 5 min. Then the mixture was incubated at 37°C for 1 h by adding 1 μL Plasmid-Safe™ ATP Dependent DNase (Epicentre Biotechnologies) and 1 μL ATP solution (25mM, Epicentre Biotechnologies), to digest contaminating linear DNA. Then 1 μL of Golden Gate reaction mix was transformed to 30 μL TOP10 Competent Cell by electro-transformation procedure, followed by adding 300 μL of liquid LB to the transformation mix, and cells were recovered for 40 min at 37 °C under agitation. Then 150 μL of the transformation mix was plated into LB agar plates containing kanamycin or Ampicillin and incubated overnight at 37°C. Positive clones were confirmed by Colony PCR and sequencing.

Bacterial Strains

Escherichia coli TOP10 was used for cloning and plasmid propagation. E. coli strain DB3.1 was used for the generation or proliferation of recipient vectors that carry the *ccdB* gene.

Generation of Transgenic Lines

All transgenic lines in this paper were generated by co-injected the final constructs with *I-SceI* meganuclease (NEB) or *Tol2* transposase RNA into zebrafish embryos of the AB genetic background at the one-cell stage as previously described (Grabher et al., 2004).

Heat-shock treatment

Embryos at the specific stage were transferred to a water bath at 38.5 °C for 40 min to induce protein expression, then return to 28.5°C for further raising. Embryos were screened for fluorescent protein expression and imaged by a confocal microscope.

Confocal microscopy

Multispectral fluorescence microscopy was performed on an LSM 880 confocal microscope (Carl

Zeiss). Images were then loaded into ZEN Connect (Carl Zeiss) and composed into a single multi-channel image with 8-bit.

Supplementary References

Chen, J., He, J., Ni, R., et al. (2019). Cerebrovascular Injuries Induce Lymphatic Invasion into Brain Parenchyma to Guide Vascular Regeneration in Zebrafish. Dev Cell *49*, 697-710 e695.

Chen, J., Li, X., Ni, R., et al. (2021). Acute brain vascular regeneration occurs via lymphatic transdifferentiation. Dev Cell *56*, 3115-3127 e3116.

Cohen, S.N., Chang, A.C.Y., Boyer, H.W., et al. (1973). Construction of Biologically Functional Bacterial Plasmids in-Vitro. P Natl Acad Sci USA *70*, 3240-3244.

Engler, C., Gruetzner, R., Kandzia, R., et al. (2009). Golden gate shuffling: a one-pot DNA shuffling method based on type IIs restriction enzymes. PLoS One *4*, e5553.

Engler, C., Kandzia, R., and Marillonnet, S. (2008). A one pot, one step, precision cloning method with high throughput capability. PLoS One *3*, e3647.

Fonseca, J.P., Bonny, A.R., Kumar, G.R., et al. (2019). A Toolkit for Rapid Modular Construction of Biological Circuits in Mammalian Cells. ACS Synth Biol *8*, 2593-2606.

Geu-Flores, F., Nour-Eldin, H.H., Nielsen, M.T., et al. (2007). USER fusion: a rapid and efficient method for simultaneous fusion and cloning of multiple PCR products. Nucleic Acids Res *35*, e55.

Gibson, D.G., Young, L., Chuang, R.Y., et al. (2009). Enzymatic assembly of DNA molecules up to several hundred kilobases. Nat Methods *6*, 343-345.

Grabher, C., Joly, J.S., and Wittbrodt, J. (2004). Highly efficient zebrafish transgenesis mediated by the meganuclease I-SceI. Methods Cell Biol *77*, 381-401.

Hartley, J.L., Temple, G.F., and Brasch, M.A. (2000). DNA cloning using in vitro site-specific recombination. Genome Res *10*, 1788-1795.

He, J., Lu, H., Zou, Q., et al. (2014). Regeneration of liver after extreme hepatocyte loss occurs mainly via biliary transdifferentiation in zebrafish. Gastroenterology *146*, 789-800 e788.

Kozak, M. (1987). An analysis of 5'-noncoding sequences from 699 vertebrate messenger RNAs. Nucleic Acids Res *15*, 8125-8148.

Liu, C., Wu, C., Yang, Q., et al. (2016). Macrophages Mediate the Repair of Brain Vascular Rupture through Direct Physical Adhesion and Mechanical Traction. Immunity *44*, 1162-1176.

Mitchell, L.A., Chuang, J., Agmon, N., et al. (2015). Versatile genetic assembly system (VEGAS) to assemble pathways for expression in S. cerevisiae. Nucleic Acids Res *43*, 6620-6630.

Pan, Y.A., Freundlich, T., Weissman, T.A., et al. (2013). Zebrabow: multispectral cell labeling for cell tracing and lineage analysis in zebrafish. Development *140*, 2835-2846.

Patron, N.J., Orzaez, D., Marillonnet, S., et al. (2015). Standards for plant synthetic biology: a common syntax for exchange of DNA parts. New Phytol *208*, 13-19.

Roberts, R.J. (2005). How restriction enzymes became the workhorses of molecular biology. Proc

Natl Acad Sci U S A *102*, 5905-5908.

Sleight, S.C., Bartley, B.A., Lieviant, J.A., et al. (2010). In-Fusion BioBrick assembly and re-engineering. Nucleic Acids Res *38*, 2624-2636.

Weber, E., Gruetzner, R., Werner, S., et al. (2011). Assembly of designer TAL effectors by Golden Gate cloning. PLoS One *6*, e19722.

Yang, Y., Wang, H., He, J., et al. (2021). A single-cell-resolution fate map of endoderm reveals demarcation of pancreatic progenitors by cell cycle. Proc Natl Acad Sci U S A *118*.

Zhong, Y., Huang, W., Du, J., et al. (2019). Improved Tol2-mediated enhancer trap identifies weakly expressed genes during liver and beta cell development and regeneration in zebrafish. J Biol Chem *294*, 932-940.
